# Can the Suspension Method (Tenderstretch vs. Achilles Tendon) Enhance Horsemeat Quality?

**DOI:** 10.3390/ani14233540

**Published:** 2024-12-07

**Authors:** Ana Kaić, Barbara Luštrek, Silvester Žgur, Klemen Potočnik

**Affiliations:** 1 Department of Animal Science and Technology, Faculty of Agriculture, University of Zagreb, Svetošimunska 25, 10000 Zagreb, Croatia; 2 Department of Animal Science, Biotechnical Faculty, University of Ljubljana, Jamnikarjeva 101, 1000 Ljubljana, Slovenia; barbara.lustrek@bf.uni-lj.si (B.L.); silvo.zgur@bf.uni-lj.si (S.Ž.); klemen.potocnik@bf.uni-lj.si (K.P.)

**Keywords:** hanging method, horse, meat quality, muscles

## Abstract

This study investigated whether the suspension method (tenderstretch, TS or Achilles tendon, AT) can improve the quality of horsemeat by analyzing longissimus dorsi (LD) and semitendinosus (ST) muscles. A total of 25 horse carcasses were considered experimental units and split longitudinally, with one half suspended using the TS method and the other half using the AT method, which enabled a direct comparison within the carcass. After 7 days of aging under commercial processing conditions, the LD and ST muscles were analyzed for pH, color (L*, a*, b*), water-holding capacity (drip loss, thawing loss, cooking loss), tenderness (Warner–Bratzler shear force), and sarcomere length. Statistical analysis was performed using the MIXED procedure in SAS, with Bonferroni correction applied for post hoc comparisons. Significant differences were found between the muscles: LD had higher tenderness (39.28 N vs. 49.77 N, *p* = 0.0011), lower cooking loss (23.56% vs. 27.04%, *p* = 0.0002), and higher thawing loss (12.38% vs. 9.72%, *p* = 0.0021) compared to ST muscle, which had a lighter color (L* = 41.90 vs. 37.73, *p* < 0.0001) and longer sarcomeres (2.22 μm vs. 1.74 μm, *p* < 0.0001). While the TS suspension method significantly increased sarcomere length (2.05 μm vs. 1.92 μm, *p* = 0.0020), it did not lead to significant improvements in other quality attributes such as pH, water-holding capacity, or tenderness. The results indicate that although the TS method affects muscle structure by elongating sarcomeres (with an average difference of 0.13 μm), it does not significantly improve the overall quality of the horsemeat compared to the AT method after 7 days of aging. A combination of factors beyond suspension methods, such as optimizing aging periods or considering additional processing techniques, may therefore be required to improve horsemeat quality. This study provides insights into the specific attributes of LD and ST muscles and their response to suspension methods and contributes to a better understanding of optimizing horsemeat quality for commercial purposes.

## Simple Summary

This study investigated whether different ways of hanging horse carcasses after slaughter, known as tenderstretch (pelvic suspension) and Achilles tendon suspension, could improve the quality of horsemeat after 7 days of aging. The researchers focused on the *longissimus dorsi* and semitendinosus muscles and analyzed pH, color, water-holding capacity, and sarcomere length. The tenderstretch method resulted in a slight lengthening of the muscle fibers, often associated with improved tenderness. However, other quality attributes such as tenderness, water-holding capacity, or color were not significantly improved. The longissimus dorsi muscle was more tender and retained less water during cooking than the semitendinosus muscle, which was tougher and had a lighter color. The results suggest that although the tenderstretch method affects muscle structure, it may not be enough to significantly improve the overall quality of the meat on its own. Additional factors, such as aging processes or other techniques, may be required to improve the quality of horsemeat further. This study provides useful insights into the horsemeat industry and helps producers to better understand how to optimize meat quality from a consumer point of view.

## 1. Introduction

In recent years, horsemeat has become increasingly popular in some parts of the world and has great potential as an alternative meat for human consumption. According to the latest data from FAOSTAT, horsemeat production accounts for about 0.21% of total global meat production [1]. The purchase and consumption of horsemeat is determined by consumer habits, which largely depend on the country or region [2]. Estimated production by continent for 2022 shows that 57.2% of horses are produced for horsemeat in Asia, 25.4% in the Americas, 11.9% in Europe, 3.2% in Oceania, and 2.2% in Africa [1]. The estimated global production of horsemeat in 2022 was 775,543.24 t, of which 92,571.46 t were produced in Europe [1].

It has been shown that attitudes towards horsemeat have changed and consumers are interested in “environmentally friendly” meat and even more in a healthy nutritional meat profile [3,4,5]. In this context, the digestive physiology of equines compared to ruminants allows for an efficient absorption of n-3 polyunsaturated fatty acids from pasture into meat, with a lower deposition of trans-fatty acids and methane emissions produced per unit of meat [6]. From a nutritional point of view, horsemeat is a good source of important nutrients, characterized by a high content of proteins and trace elements (iron and B-group vitamins), a low fat content, and a favorable fatty acid profile with high concentrations of polyunsaturated fatty acids and a low cholesterol content [5,6,7,8,9]. Furthermore, it has been pointed out that horses are very suitable for meat production, which is reflected in the excellent dressing percentages and high lean meat yield in the carcass. De Palo et al. [10] reported dressing percentage as high as 73.71% and 78.96% lean meat in the carcass in Italian Heavy Draught horse foals.

In terms of quality attributes, horsemeat is not inferior to other types of meat valued by consumers [5]. Color is an important attribute that consumers associate with freshness and quality, and they prefer to buy red meat over brown meat based on visual judgment [11]. However, horsemeat is recognized by its dark color, which turns brown/black with a bluish tinge when exposed to air. It is rich in myoglobin and has a high ability to combine with oxygen, so that oxidation is accelerated, i.e., the bright red color is transformed (oxidized) into a brown color. To meet consumer expectations, producers try to improve color stability through various methods, e.g., appropriate packaging, storage techniques, etc. [12].

Horsemeat exhibits a relatively rapid pH decline after slaughter and is characterized by a relatively good water-holding capacity (WHC) [13,14,15]. A higher WHC is more appreciated by consumers (better visual appeal due to lower drip losses) and enables the use of horsemeat in semi-product technology by reducing moisture loss during thermal treatment and thus increasing the yield of finished products [15,16].

Compared to a beef carcass, horses have a higher proportion of easily trimmed subcutaneous and body cavity fat and a lower proportion of intermuscular and intramuscular fat [17]. Due to the low proportion of intramuscular fat and its low melting temperature, the juiciness of horsemeat does not differ significantly from that of other types of meat [13,14]. Horsemeat is recognizable by its sweet taste, which is mainly due to its high glycogen content [18].

According to Kim and Joo [19] and Stanislawczyk et al. [5], the horsemeat obtained after slaughter is relatively tough, cohesive, and has an unfavorable tenderness. The insufficient tenderness is due to the high content of connective tissue proteins and the higher thermal resistance of the horse’s muscle collagen [20,21]. However, the literature reports the Warner–Bratzler shear force (WBSF) value for horsemeat to be between 2.1 kg/cm^2^ [9] and 7.7 kg/cm^2^ [18]. The tenderness of horsemeat can therefore range from ‘very tender’ to ‘tough’. Previous studies confirm that consumers can determine differences in meat tenderness and are willing to pay a higher price for meat if they perceive it to be more tender [4,22].

Today, the meat industry is faced with the challenge of meeting consumer expectations in terms of meat quality, which is directly linked to retaining existing consumers and attracting new ones, increasing their frequency of consumption, and thus ensuring a higher selling price. Therefore, the meat industry makes great efforts to eliminate unfavorable properties of horsemeat (e.g., color and tenderness) immediately after slaughter through various interventions (mechanical, chemical, biochemical, physical, and enzymatic) [5,19]. Physical interventions are those that induce structural changes in the meat through the application of force or physical stimuli, with the result being visible as changes in connective tissue, myofibrillar protein networks, and the stimulation of proteolysis through direct interactions between endogenous enzymes, cofactors, and substrates [23]. In brief, tenderstretch is a physical method (also known as hip suspension or pelvic suspension) in which carcasses are suspended by the pelvic bone, causing the hind leg to hang more horizontally. This suspension method results in a more natural position of the muscles in relation to the carcass [24,25]. It increases the tension of the loin and hindquarter muscles during rigor establishment, avoiding intense contractions and making the muscles more tender [26]. Previous studies have shown that tenderstretch has a large effect on the palatability of the hind limb and loin muscles of the carcass, while there is no effect on the forequarter muscles [24,25,27]. The positive effect of tenderstretch on the meat tenderness has already been demonstrated in the M. longissimus dorsi, *M. semimembranosus*, *M. gluteus medius*, and *M. adductor* of cattle [28,29,30], the *M. semimembranosus* and *M. longissimus thoracis* et lumborum of lambs [31,32] and pigs [33], as well as the M. semimembranosus of alpacas [34].

To our knowledge, no studies have yet been conducted on the effect of the suspension method on horsemeat quality. The aim of this experiment was therefore to investigate the effect of the suspension method (tenderstretch vs. Achilles tendon) on the pH value, color parameters, water-holding capacity, tenderness assessed by WBSF, and sarcomere length of two muscles (M. longissimus dorsi and M. semitendinosus) in horse carcasses under commercial conditions.

## 2. Materials and Methods

### 2.1. Animals, Slaughtering Procedure, and Sample Collection

As in routine practice, the animals were processed on different days according to their arrival at the accredited abattoir. A total of 25 horses (9 females and 16 males) of different breeds (Slovenian draft horse; n = 4, Posavje horse; n = 5, Trotter; n = 1 and crossbreeds; n = 15) were involved in this study. The age of the animals involved in the study ranged from 8 months to 15 years, with a median of 12 months and an average age of 28 months. Each horse carcass served as an experimental unit and was split longitudinally into two halves to allow for a direct comparison of the two suspension methods within the carcass.

**Figure 1:**
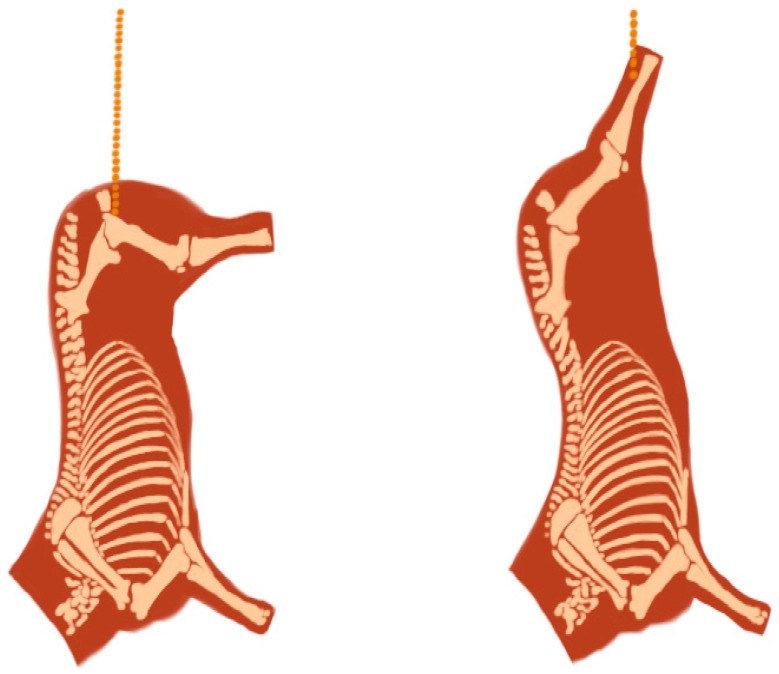
Schematic illustration of the different suspension methods. The carcass shapes resulting from the tenderstretch method (**left**) and from the Achilles tendon method (**right**).

The animals were stunned in the frontal head area with a captive bolt stunner, slaughtered, and trimmed by the current EU regulations [35]. Before entering the chilling room, the carcasses were divided longitudinally into two halves. One half of each carcass was suspended using the conventional method (Achilles tendon; AT), where the hanging hook is positioned through the hind heels, while the other side of the carcass was suspended by the pelvic bone (tenderstretch; TS; see Figure 1). The TS method consisted of the following procedure: the half-carcasses were lifted using a manual tackle and an S-shaped hook was inserted into the foramen obturator so that the hind limb hung in a horizontal position of approximately 90° to the carcass. According to commercial practice, the carcasses were placed in a refrigerated chamber at 4 °C and aged for 7 days. After the aging period, the M. longissimus dorsi (LD) and M. semitendinosus (ST) were removed from each half-carcass. The samples were then immediately transported to the laboratory of the Department of Animal Science at the Biotechnical Faculty (University of Ljubljana, Slovenia) for further analysis.

The LD and ST samples were initially used for pH and color measurements (L*, a*, b*). Then, a 1 cm thick slice was cut for drip loss (DL), while two 2 cm thick slices were vacuum packed and frozen at −20 °C until further analyses were performed. After the storage period, the LD and ST samples were thawed and used to determine thawing loss (TL), cooking loss (CL), WBSF, and sarcomere length.

### 2.2. Analytical Methods

The pH value of the samples was determined using a portable pH meter (seven2 go, Mettler Toledo, Greifensee, Switzerland) equipped with a penetration glass probe. Before the measurements were made, the device was calibrated with buffer solutions with pH values of 4.00 and 7.00 (Mettler Toledo, Greifensee, Switzerland). Muscle color parameters were measured in triplicate on cross-sections of the LD and ST muscles after a 1 h blooming period using a Minolta CR-300 (Konica Minolta Sensing Inc., Osaka, Japan) chroma meter (D65 standard illuminant, Ø8 mm measuring area, and 0° viewing angle). Before the measurements, the chroma meter was calibrated using a standard white ceramic tile (Y = 93.6, x = 0.3129 and y = 0.3193). The color was expressed according to the CIE Lab system, measuring lightness (L*), redness (a*), and yellowness (b*) [36]. Three measurements were made for each sample, the mean value of which was used for further analysis.

The water-holding capacity was measured as DL, TL, and CL, and determined on LD and ST muscle samples according to the method described by Honikel [37]. To determine the DL, the 1 cm thick slice of the muscle sample was dried with paper towels, weighed, and then hung in a polyethylene bag, taking care that there was no contact between the sample and the bag. After 24 h of storage at 4 °C in the refrigerator, the samples were removed from the bags, dried with paper towels, and weighed again. The DL was expressed as the percentage of weight loss after the suspension of the sample in relation to the initial weight of the sample.

For TL determination, the frozen and vacuum-packed meat samples were thawed in the refrigerator (at 4 °C) for 24 h. The thawed, 2 cm thick samples were removed from the bags, dried with paper towels, and weighed again. The TL was expressed as the percentage weight loss of the thawed sample compared to the initial sample weight.

For CL determination, the samples were weighed, placed in vacuum bags, and cooked in a preheated water bath (85 °C) until they reached an internal temperature of 75 °C, which was monitored with thermocouples connected to a data logger (TFN 530 Thermometer, Xylem Analytics Germany Sales GmbH & Co. KG, Waldheim, Germany). After cooking, the samples were cooled under running water for 20 min, cooled to equilibrium, dried with paper towels, and weighed again. The CL was calculated as the difference in weight between the cooked and raw samples and expressed as a percentage of the initial weight before cooking.

After CL measurement, WBSF determination was performed using an Instron Universal Testing Machine (Model 3345, Instron, Canton, MA, USA) calibrated to full scale with a 500 Newton load cell, a crosshead speed of 250 mm/min, and a sampling rate of 10 points/s. The eight square cores (1 × 1 × 1.5 cm) of each sample were cut parallel to the muscle fibers and sheared once perpendicular to the longitudinal axis of the fibers using a Warner–Bratzler V-shaped blade. The WBSF was measured as the mean value of the eight peak force recordings on the muscle cores of each sample and expressed in Newtons.

The samples for measuring sarcomere length were prepared as described by Hegarty and Naudé [38]. For this purpose, the muscles were frozen in a cryostat (Leica CM1850, Nussloch, Germany). An approx. 200 µm thick slice was cut, removed, and homogenized in 1 mL Ringer’s solution using a laboratory blender (T 18 basic ULTRA-TURRAX, IKA, Staufen, Germany). A small drop of the homogenized sample was observed with a microscope (Leica DM750, PR, Leica Microsystems GmbH, Wetzlar, Germany) equipped with a camera (Leica ICC50 HD, Leica Microsystems GmbH, Wetzlar, Germany). Within each prepared sample, the photos of 10 myofibers were taken from the randomly selected areas. The Micro-manager V 2.0. program was used to determine sarcomere length. Within each myofiber, the sarcomere length was measured on 10 myofibrils, and a median value of one hundred measurements was used for further analysis.

### 2.3. Statistical Analyses

A variance analysis of the meat quality traits was performed using the MIXED procedure of the SAS/STAT software package (v9.4 SAS Institute Inc., Cary, NC, USA). The best fitted model for pH value, color parameters (L* a* b*), DL, TL, CL, WBSF, and sarcomere length was as follows:

$$y_{ijk}=\mu +M_{i}+S_{j}+e_{ijk}$$

and included the overall mean (*μ*), muscle type (*i* = LD, ST), and suspension method (*j* = TS, AT) as fixed effects, and the random residual (*e_ijk_*). The interaction between the suspension method and muscle type was not statistically significant for the meat quality traits (*p* ≥ 0.05) and was excluded from the model and further analysis. A post hoc comparison between the least square means was performed with a Bonferroni multiple test correction. Differences were considered at significance level *p* < 0.05.

## 3. Results and Discussion

### 3.1. pH Value

The pH value of horsemeat is an important meat quality trait that influences several important characteristics such as color, water-holding capacity, tenderness, juiciness, and shelf life, i.e., it determines the technological and culinary usability of meat [39]. The results showed that with pH values ranging from 5.75 to 5.85, the acidification process remained within an acceptable range. However, further analysis revealed significant differences in pH values between LD and ST muscles (*p* = 0.0007; Table 1). Seong et. [12] also observed variations in pH values between different retail cuts of horsemeat, with a shoulder chuck roll having the highest value (5.95), followed by the shank (5.85), the loin (5.80), shoulder clod (5.74), and short plate brisket (5.70), while the lowest values were found for the strip loin (5.58), top round (5.60), outside round (5.62), and brisket (5.66). Kaić et al. [40] also found significant differences between LD (5.52) and ST (5.61) muscles of horsemeat. Seong et al. [12] and Kaić et al. [40] attributed these differences in pH values to factors such as the physical activity of the muscles, glycogen storage levels, and the rate of degradation. In contrast, Litwińczuk et al. [18] found that pH values stabilized 48 h after slaughter and that there was no significant difference between LD (5.67) and ST (5.68) muscles. Tateo et al. [41] found no significant differences in pH values measured 72 h after slaughter between the biceps femoris (BF; 5.65), LD (5.56), rectus femoris (RF;5.77), semimembranosus (SM; 5.61), and ST (5.61) muscles. Franco and Lorenzo [42] also confirmed that the pH values showed no significant differences between different muscles (triceps brachii (TB), psoas major and minor (PM), LD, SM, ST, and BF) and ranged between 5.61 and 5.69. These contrasting results can be attributed to different conditions of the animals before slaughter (e.g., stress level and duration of fasting), feeding or diet, and age at slaughter in the different studies [12,40].

No significant differences in pH values were found between the suspension methods (*p* = 0.8138; Table 1). Similarly, Channon et al. [33] found no differences in the ultimate pH of pork between different suspension methods (Achilles vs. Aitchbone). Pinheiro and Souza [32] also reported that the pH of the meat was not affected by the suspension method (Gastrocnemius vs. Aitchbone) of a sheep carcass. Luchiari Filho et al. [43] observed no difference in meat pH values with different suspension methods (Achilles vs. carpi radialis muscle) of Nellore steer carcasses. Ahnstrom et al. [29] found no differences in the pH values of the meat with different suspension methods (TS vs. AT) in beef carcasses. The lack of significant differences is probably due to the fact that pH is primarily determined by the postmortem biochemical process of glycolysis, which is consistent across muscles and is largely unaffected by the external suspension method. Suspension methods do not appear to affect glycogen depletion or lactic acid production, which are the main factors in the pH decline.

### 3.2. Color Parameters

The color of meat is an important trait for the meat industry, as it strongly influences the consumers’ perception of product quality and thus also has a significant influence on the purchase decision [44]. A comparison of the colorimetric values revealed a significant difference (*p* < 0.0001) in lightness (L*) between the muscles, with the ST muscle being lighter (with higher L* values) than the LD muscle (Table 1). This result is consistent with the reports of Tateo et al. [41], Lorenzo et al. [45], and Kaić et al. [40], who also observed that the ST muscle was lighter than the LD muscle. Specifically, Tateo et al. [41] reported an L* value of 37.73 for the ST muscle and 36.58 for the LD muscle; Lorenzo et al. [45] found L* values of 40.86 for the ST muscle and 39.16 for the LD muscle; and Kaić et al. [40] found L* values of 44.62 for the ST muscle and 39.76 for the LD muscle. In addition, Tateo et al. [41] and Lorenzo et al. [45] found that the ST muscle was lighter than the SM and TB muscles. The results for meat redness (a*) showed a significant difference between the muscles (*p* = 0.0055). Tateo et al. [41] also found significant differences in the a* values between muscles, suggesting that the LD (11.34), SM (11.62), and RF (11.92) muscles had a more intense red color (higher a* values) than the ST (10.70) and BF (9.38) muscles. However, Lorenzo et al. [45] and Kaić et al. [40] found no significant differences in a* values between the LD and ST muscles. Finally, the results for the yellowness (b*) showed significantly higher values in the ST muscles (*p* < 0.0001). Kaić et al. [40] also found significantly higher b* values in the ST (11.55) muscle than in the LD (10.25) muscle, while Lorenzo et al. [45] and Tateo et al. [41] did not find significant differences in b* values between the ST and LD muscles.

The colorimetric parameters of the investigated horsemeat were not significantly influenced by the suspension method (*p* > 0.05; Table 1). This could be because the meat color depends primarily on the concentration and oxidation state of myoglobin, which are not affected by the stretching of the muscles during hanging. In addition, pH, a critical factor for color stability, showed no significant differences between the suspension methods, which further explains the lack of color differences. Similarly, Baldassini et al. [46] and Bayraktaroglu and Kahraman [47] reported that the suspension methods (TS vs. AT) of beef carcasses had no significant effect on meat color parameters (*p* > 0.05). Channon et al. [33] also found no significant differences in the color parameters of pork between the different suspension methods (Achilles vs. Aitchbone). Similarly, Pinheiro and Souza [32] found that the color parameters of carcasses of discarded ewes, suspended by the gastrocnemius tendon or the pelvic bone, did not differ significantly (*p* > 0.05).

### 3.3. Drip Loss

The quality of the meat is significantly influenced by its WHC, which is essential for both processing technology and consumer acceptance. A poor WHC can lead to the excessive dripping of meat and meat products, resulting in significant weight loss in carcasses and cuts. This in turn has a negative impact on the yield and quality of fresh and processed meat, making it less attractive to consumers [48]. As a key indicator of WHC, DL is considered one of the most important parameters for the assessment of meat quality [49]. The results of this study show that muscle type has no significant influence on DL (*p* = 0.8717; Table 2). Similar results with non-significant differences between LD and ST muscles were reported by Kaić et al. [40] and between LD, ST, SM, BF, TB, and PM muscles by Franco and Lorenzo [42]. However, it is noteworthy that Kaić et al. [40] reported slightly lower DL values for the LD (1.51%) and ST (1.65%) muscles compared to our values, which could be attributed to differences in animal breeds, processing methods, or aging conditions. Similarly, Franco and Lorenzo [42] observed comparable DL values for the LD (1.83%) and ST (1.93%) muscles, but reported slightly lower DL values for the SM (1.74%), BF (1.79%), and TB (1.71%) muscles and a higher DL value for the PM muscle (1.99%) compared to our results. These discrepancies illustrate that DL may be influenced by several factors, including muscle fiber composition, postmortem treatment, and environmental conditions, and not only by muscle type.

The DL values of horsemeat were not significantly affected by the suspension method (*p* = 0.1951; Table 2). However, it is worth noting that TS-suspended carcasses had a lower DL compared to those suspended by the AT. Similarly, Ahnström et al. [29] found that the TS suspension method resulted in lower, though not statistically significant, DL values in the LD muscle of Swedish Red cattle carcasses. Baldassini et al. [46] observed the same trend in the LD muscle of Brangus heifers and Nellore bulls. Bayraktaroglu and Kahraman [47] found similar results for the BF muscle. In contrast, many studies have shown that the TS suspension method significantly improves WHC by reducing DL in various muscles under conventional chilling [29,30,33,50,51]. Marsh et al. [52] highlighted that DL is influenced by the shrinkage of myofibrils during *rigor mortis*. Pelvic suspension causes the vertebral column and surrounding muscles to straighten and stretch more compared to the AT suspension method, resulting in higher water retention and lower DL [53].

### 3.4. Thawing Loss

The freezing and subsequent thawing of meat can significantly affect its quality characteristics, including moisture retention, protein denaturation, oxidation, color, and texture, and ultimately reduce consumer acceptance [54]. The results of the present study show that the LD muscle had a significantly higher TL value than the ST muscle (*p* = 0.0021; Table 2). These TL values are consistent with our previous results [40], where the LD muscle (8.99%) also had a significantly higher TL than the ST muscle (8.00%). However, the previous study showed lower TL values compared to the current results. Tateo et al. [41] also reported slightly higher TL values in the LD muscle (5.81%) compared to the ST muscle (5.69%), but these were not statistically significant. Kim and Brad Kim [55] found that each stage of aging, freezing, and thawing, whether individually or in combination, can affect different quality characteristics of meat. Different aging, freezing, and thawing conditions could be responsible for the observed differences in TL. In our previous study [40], samples were aged for 14 and 28 days, while in the current study, they were aged for 7 days, keeping the other procedures (vacuum packaging, storage until the end of the experiment, and thawing at 4 °C for 24 h) the same. Tateo et al. [41] vacuum packaged their samples 72 h postmortem, stored them for 10 days, and thawed them at 2 to 5 °C for 24 h. In agreement with the results of DL findings, the TS suspension method resulted in lower TL values than the AT suspension method, but this difference was not statistically significant (*p* = 0.3525; Table 2). The lack of statistical significance in TL suggests that while TS may provide a slight benefit in reducing TL, the effect may be too small to be consistently measurable under the conditions of this study. Factors such as individual variation between carcasses, differences in muscle sensitivity to suspension methods or the relatively short aging period (7 days) may have contributed to these results.

Ahnström et al. [28] observed significant differences in TL for the adductor, SM, and LD muscles of Charolais heifers after 7 and 14 days of aging, with the TL values being lower for carcasses suspended using the TS method compared to the AT method. Furthermore, it was noted that TL decreased with extended aging duration, indicating that prolonged aging may enhance water retention in certain muscles.

### 3.5. Cooking Loss

The cooking yield provides crucial information about the properties of the raw muscle protein and the functionality of the meat, influencing the yield and quality of the further-processed products [56]. The present study showed significant differences in CL values between muscles, with ST muscle (27.04%) showing greater losses than LD muscle (23.56%; *p* = 0.0002; Table 2). Similarly, Kaić et al. [40] reported a significant difference in CL between the ST (24.13%) and LD (19.52%) muscles, although their values were lower than those found in the present study. These differences could be related to different aging periods; in our previous study, samples were aged for 14 and 28 days, whereas in the present study, aging was limited to 7 days, which may gradually affect WHC over time. In contrast, Tateo et al. [41] found no significant differences in CL between the LD (25.4%) and ST (26.21%) muscles, although their values were higher than those previously reported. These differences could be due to different cooking conditions affecting the final cooking yield [57]. Tateo et al. [41] measured CL when the internal temperature of the sample reached 70 °C and was held for 3 min, whereas in our two studies mentioned above, CL was measured at an endpoint temperature of 75 °C.

In the present study, CL values were not significantly affected by the suspension method (*p* = 0.9190; Table 2). Previous studies on CL values influenced by the TS vs. AT suspension method have shown considerable variability. The studies showed different results regarding CL values after TS suspension, with results indicating no effect on CL [30,51], an increase in CL [46,58], and a decrease in CL [43,50]. These differences could be due to the different methods of hanging carcasses, the muscles analyzed, the methods used in analyzing CL or even the animal species used [43].

### 3.6. Warner–Bratzler Shear Force

**Figure 2:**
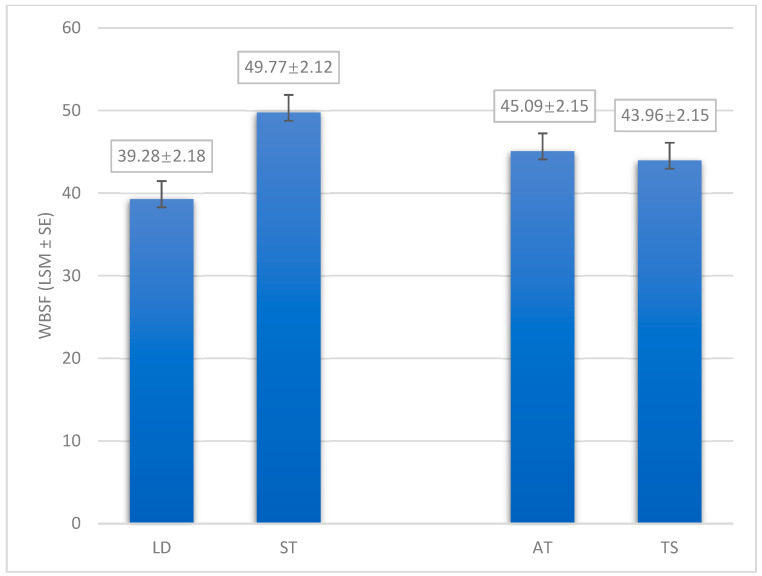
The effect of muscle and suspension method on the Warner–Bratzler shear force (LSM ± SE). LT = longissimus dorsi muscle; ST = semitendinosus muscle; AT = Achilles tendon method, TS = tenderstretch method; WBSF = Warner–Bratzler shear force.

Tenderness is another important and complex quality attribute that influences consumer acceptance. Consumers can recognize differences in the tenderness of meat and are willing to pay a higher price if they perceive the meat to be more tender [59]. The present study shows a significant difference between the LD muscle and the ST muscle in WBSF values (*p* = 0.0011), indicating that the LD muscle has greater tenderness (39.28 N) compared to the ST muscle (49.77 N; Figure 2). This result is in agreement with Kaić et al. [40], who also found significantly higher tenderness in the LD muscle (25.7 N) compared to the ST muscle (47.22 N). Tateo et al. [41] investigated the WBSF differences between five different muscles (BF, LD, RF, SM, ST) and found that the ST muscle was the most tender (5.33 kg) while the BF muscle was the toughest (5.95 kg). According to Franco and Lorenzo [42], the major influence of muscle type on tenderness can be explained by the different physiological functions of muscles in relation to their anatomical location, which include different histological traits such as collagen quantity and quality (solubility) and sarcomere length.

Although the suspension method had no significant effect on WBSF values, the TS method showed lower values (43.96 N), indicating greater tenderness compared to the AT method (45.10 N; *p* = 0.7097; Figure 2). The reduction in WBSF between the AT and TS methods was only 1.14 N after 7 days of aging. The optimal aging period for foal meat is not yet defined, and the usual practice of butchers specializing in horsemeat is to market it after 4 days of aging [60]. In our study, it was common practice in the processing plant to market horsemeat after 7 days of aging. Liu et al. [30] found that the WBSF values of Chinese Yellow cattle carcasses in LD muscle were significantly reduced by the TS suspension method after 1 day of aging. After 7 and 14 days of aging, the WBSF values of LD muscle suspended with the TS method were also lower than those with the AT method, but the difference was not significant. Similar results were reported by Bayraktaroglu and Kahraman [47] for the BF muscle of Holstein × Friesian crosses. Derbyshire et al. [61] also found lower but not significantly different WBSF values after 7 days of aging in the LD muscle of Bonsmara steer carcasses suspended with the TS method, although a significant decrease was not observed until 24 h post-slaughter. Thus, while the TS suspension method showed a trend to improve tenderness, its effect on WBSF becomes less pronounced with longer aging, suggesting that the benefits of TS suspension may be more pronounced with shorter aging period. Further studies are required to investigate the optimal aging period for foal meat and to determine the most effective combination of the suspension method and aging period to improve meat tenderness.

### 3.7. Sarcomere Length

**Figure 3:**
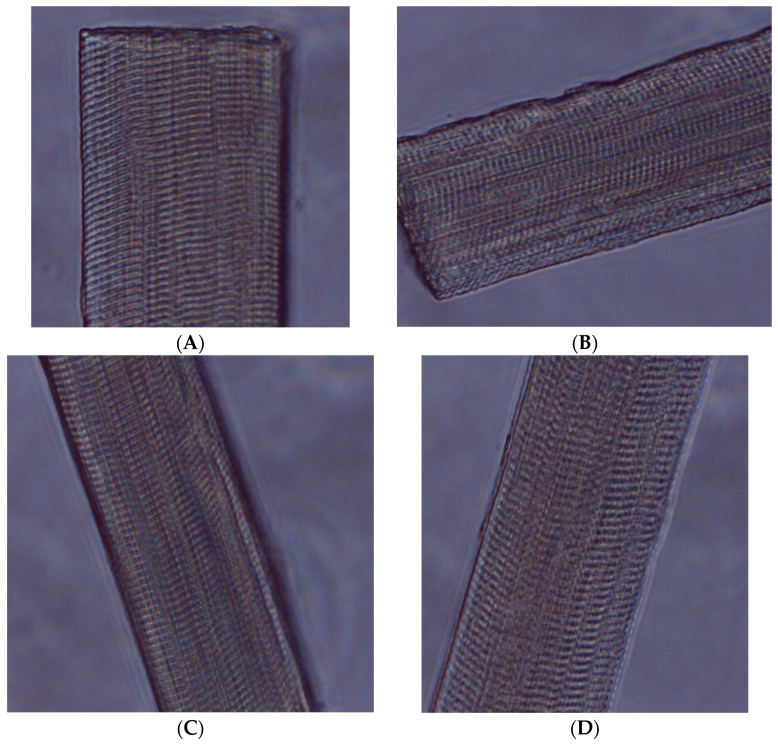
Transmission light micrographs of sarcomere lengths in horsemeat: (**A**) LD muscle suspended using the Achilles tendon method, (**B**) ST muscle using the Achilles tendon method, (**C**) LD muscle using the tenderstretch method, and (**D**) ST muscle using the tenderstretch method.

**Figure 4:**
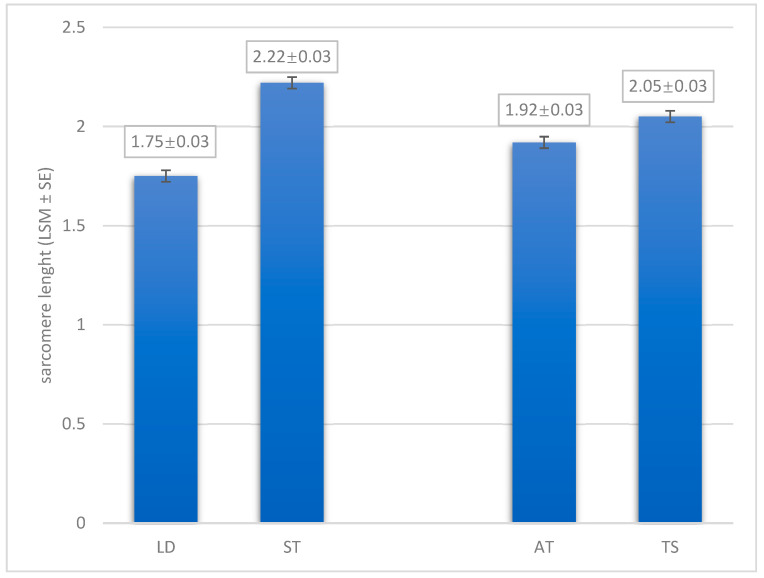
The effect of muscle and suspension method on the sarcomere length (LSM ± SE). LD = longissimus dorsi muscle; ST = semitendinosus muscle; AT = Achilles tendon method, TS = tenderstretch method.

Postmortem sarcomere lengths have a significant effect on the texture of raw and cooked meat, directly influencing the WHC of raw meat, and indirectly affecting the color, taste, and other meat quality attributes [25]. Transmission light micrographs (Figure 3) reveal variations in sarcomere length between LD and ST muscles, as well as variations in suspension methods. The further analysis of the results show a significant difference between LD and ST muscles in sarcomere length (*p* < 0.0001; Figure 4). The estimated difference in sarcomere length between the muscles shows that the ST muscle is 0.48 μm longer than the LD muscle. Sarcomere length decreases with muscle contraction, and following rigor, significant variation in sarcomere length can occur both within and between muscles [25]. Locker [62], for example, found that sarcomere lengths in different bovine muscles varied between 0.7 and 3.7 µm after rigor.

Furthermore, the relationship between improved tenderness and increased sarcomere length shows that as sarcomere length increases, the shear force decreases [47,51,63], even at 14 days postmortem, as demonstrated by Rhee et al. [64] in bovine LD muscle (stored at 2 °C for 14 days and then frozen at −30 °C). However, Roy et al. [65] found no significant correlation between sarcomere length and WBSF, suggesting that sarcomere length does not significantly influence the tenderness of horsemeat. This result is consistent with previous studies, such as Wheeler et al. [66] on pork and Tschirhart-Hoelscher et al. [67] on lamb SM muscle, in which sarcomere length also did not affect meat tenderness.

The results of the suspension method on the sarcomere length of horsemeat are shown in Figure 4. The TS method significantly increased sarcomere length (*p* = 0.0020) compared to the AT suspension method, with an average difference of 0.13 μm. Similar results were reported by Hou et al. [51] and Liu et al. [30], who observed that pre-rigor mortis muscle stretching by the TS method significantly increased sarcomere length compared to the AT method. Specifically, Liu et al. [30] found that sarcomere length increased by 0.38 μm in the LD muscle of Chinese Yellow cattle carcasses after 7 days of aging, while Hou et al. [51] reported an increase of 0.36 μm in the LD muscle of Chinese fattened-cattle carcasses after the same aging period. This elongation is likely due to the muscle being more elongated upon entering rigor mortis and retaining this altered shape, which could be due to the effects of the TS suspension method [50].

## 4. Conclusions

The study shows significant differences between the LD and ST muscles of horse carcasses. The LD muscle was more tender and had a lower cooking loss and a higher thawing loss than the ST muscle. In addition, the ST muscle had a lighter color. These results highlight the unique structural and functional characteristics of each muscle, which significantly affect meat quality and suitability for various culinary and processing purposes.

Although the TS suspension method significantly increases the sarcomere length, it does not significantly improve other quality characteristics such as tenderness, pH, color, or water-holding capacity after 7 days of aging. This shows that improving muscle structure alone is not enough to optimize meat quality. To achieve the best results in horsemeat processing, a comprehensive approach is required that includes factors such as aging duration and other quality control measures. Shorter and longer aging periods should be investigated, as both have different effects on specific meat quality characteristics. In addition, the combination of suspension methods such as tenderstretch (TS) with other interventions (e.g., electrical stimulation or marinating) can further improve tenderness and muscle structure. Exploring the synergistic effects of these combined methods, together with different aging, should be a priority for future research to provide more specific recommendations on aging time and processing methods and to optimize horsemeat quality.

## Figures and Tables

**Table 1:** The effect of muscle and suspension method on pH value, drip loss (DL), and color parameters (L* a* b*).

	Muscle		*p*-Value	Suspension Method		*p*-Value
	LD	ST		AT	TS	
Number of samples */ half-carcass **	50 *	50 *		25 **	25 **	
pH value	5.75 ± 0.02 ^a^	5.85 ± 0.02 ^b^	0.0007	5.81 ± 0.02	5.80 ± 0.02	0.8138
L* (Lightness)	37.73 ± 0.47 ^a^	41.90 ± 0.47 ^b^	<0.0001	39.78 ± 0.47	39.84 ± 0.47	0.9264
a* (Redness)	22.80 ± 0.58 ^a^	25.13 ± 0.58 ^b^	0.0055	24.03 ± 0.58	23.90 ± 0.58	0.8679
b* (Yellowness)	11.82 ± 0.41 ^a^	14.22 ± 0.41 ^b^	<0.0001	13.21 ± 0.41	12.83 ± 0.41	0.5270

LD = longissimus dorsi muscle; ST = semitendinosus muscle; AT = Achilles tendon method, TS = tenderstretch method; LSM with different superscripts in the same row significantly differ (Bonferroni post hoc test, *p* < 0.05), * = number of samples; ** number of half-carcass.

**Table 2:** The effect of muscle and suspension method on drip loss (DL), thawing loss (TL), and cooking loss (CL).

	Muscle (M)		*p*-Value	Suspension Method		*p*-Value
	LD	ST		AT	TS	
Number of samples */half-carcass **	50 *	50 *		25 **	25 **	
DL (%)	1.83 ± 0.51	1.95 ± 0.51	0.8717	2.36 ± 0.51	1.42 ± 0.51	0.1951
TL (%)	12.38 ± 0.58 ^a^	9.72 ± 0.58 ^b^	0.0021	11.44 ± 0.58	10.66 ± 0.58	0.3525
CL (%)	23.56 ± 0.62 ^a^	27.04 ± 0.6 ^b^	0.0002	25.25 ± 0.62	25.34 ± 0.62	0.9190

LD = longissimus dorsi muscle; ST = semitendinosus muscle; AT = Achilles tendon method, TS = tenderstretch method; LSM with different superscripts in the same row significantly differ (Bonferroni post hoc test, *p* < 0.05), * = number of samples; ** number of half-carcass.

## Data Availability

The data that support the findings of this study are available from the corresponding author, A.K., upon reasonable request.
